# Myocarditis or Pericarditis Following the COVID-19 Vaccination in Adolescents: A Systematic Review

**DOI:** 10.3390/vaccines10081316

**Published:** 2022-08-15

**Authors:** Minglong Li, Xingxing Wang, Junjie Feng, Ziqi Feng, Wenwen Li, Bailiu Ya

**Affiliations:** 1 School of Basic Medical Sciences, Jining Medical University, Jining 272100, China; 2 School of Nursing, China Medical University, Shenyang 110122, China

**Keywords:** myocarditis, pericarditis, COVID-19 vaccination, adolescents

## Abstract

**Background:** By 16 May 2022, 12,186,798,032 people had been vaccinated with COVID-19 vaccines. Our study found that myocarditis/pericarditis may occur in adolescents after COVID-19 vaccination. **Methods:** In this regard, we conducted a meta-analysis of seven groups of adolescents aged 12–19 years to compare the incidence of myocarditis/pericarditis after vaccination and compare the relative risk incidence after the first and second doses of a COVID-19 vaccine, and between males and females for risk incidence. **Results:** We analyzed 22,020,997 subjects from seven studies, including 130 cases of confirmed myocarditis/pericarditis. The overall mean incidence rate was 1.69 cases per 100,000 person-years. Of these, 19 of the 12,122,244 people who received a first dose of a COVID-19 vaccine had myocarditis/pericarditis, an incidence rate of 0.0022% (95% CI 0.0001–0.0034), and 111 of the 1,008,753 people who received a second dose had myocarditis/pericarditis, an incidence rate of 0.0107% (95% CI 0.0059–0.0155). The prevalence relative ratio (RR) after the first and second doses was RR = 5.53 (95% CI: 3.01–10.16), with a higher prevalence after the second dose than after the first dose of a COVID-19 vaccine. After a second dose of a COVID-19 vaccine, the RR for males relative to females was RR = 13.91 (95% CI: 4.30–44.95), with a more pronounced risk of disease in males than in females. **Conclusions:** Our study showed that myocarditis/pericarditis occurred after vaccination with the BNT162b2 or Comirnaty vaccine, especially after the second vaccination in male adolescents, but the incidence of myocarditis/pericarditis after vaccination with the above vaccines was very rare (0.0022%). Therefore, it is recommended that adolescents should be vaccinated with the COVID-19 universal vaccine as soon as possible and closely monitored for subsequent adverse reactions, which can be treated promptly.

## 1. Introduction

The emergence of a novel coronavirus strain in 2019, severe acute respiratory syndrome coronavirus 2 (SARS-CoV-2), has led to the global spread of novel coronavirus pneumonia in 2019, and the current global pandemic remains severe. According to the latest report from the World Health Organization (WHO), as of 29 April 2022, there were 607,159 new cases and 2504 new deaths worldwide in the preceding twenty-four hours, with a cumulative total of 510 million confirmed cases, including more than 6 million deaths [1].

However, most reports related to COVID-19 have focused on adults, while several questions regarding children and adolescents with SARS-CoV-2 infection remain unanswered [2,3]. According to the American Academy of Pediatrics, the total number of reported pediatric COVID-19 cases as of 22 April 2022 was 12,937,304, with children accounting for 19.0% of all cases [4]. Currently, SARS-CoV-2 vaccination is still considered the most effective way to prevent pneumonia and reduce its mortality [5]. On 12 May 2021, the Advisory Committee on Immunization Practices (ACIP) recommended using the Pfizer-BioNTech COVID-19 vaccine in adolescents 12–15 years of age, under the U.S. Food and Drug Administration (FDA) Emergency Use Authorization (EUA) [6]. Growing evidence confirms the efficacy and safety of the SARS-CoV-2 vaccine in children and adolescents [7]. However, while affirming its safety, we have also noticed its side effects [8]. In addition to mild–moderate adverse reactions such as allergic reactions [9] and multisystem inflammatory syndrome [10] in children, we have noted more serious side effects such as myocarditis [11]. In the past few months, there have been increasing international reports of a higher, while still rare, incidence of myocarditis or pericarditis within one week of receiving the second dose of mRNA COVID-19 vaccine (Pfizer-BioNTech BNT162b2/Comirnaty), more commonly in males, mainly adolescents and young adults [4,12,13,14,15]. These adverse reactions can lead to hesitation about vaccinations [16,17].

The purpose of this paper is to collect and analyze published studies, systematically assess the incidence of myocarditis and pericarditis after COVID-19 vaccination in children and adolescents, and serve as a guide for researchers to re-evaluate and contribute to the promotion of the COVID-19 vaccine in adolescents and children.

## 2. Materials and Methods

### 2.1. Search Strategy

Our study was carried out according to the Preferred Reporting Items for Systematic Reviews and Meta-Analyses (PRISMA) [18]. The study protocol is registered in the prospective systematic review (PROSPERO, CRD42022326128).

We systematically searched for articles published in the following electronic databases: PubMed, Web of Science and Embase, for literature related to myocarditis or pericarditis after COVID-19 vaccination in adolescents. For this study, we searched using the following combinations as search terms: SARS-CoV-2 or COVID-19 or novel coronavirus, child or children or infants or adolescents or adolescence or young adults, vaccine or vaccination and myocarditis or pericarditis. Relevant literature was searched up to 1 May 2022. The detailed search formula is presented in the remainder of this section.

### 2.2. Inclusion and Exclusion Criteria

The following studies were included:

(1) Studies of the onset of myocarditis or pericarditis in adolescents following COVID-19 vaccination, including RCTs and observational studies.
(2) Age of patients ≤19 years.

The following studies were excluded:

(1) Studies not relevant to the subjects analyzed, including those that did not use the COVID-19 vaccine as an exposure parameter, and studies that were not on the onset of myocarditis or pericarditis in adolescents following vaccination.
(2) Insufficient data to calculate the incidence and outcome of myocarditis or pericarditis in adolescents with the COVID-19 vaccine.
(3) Duplication or studies with overlapping disciplines.
(4) Non-original studies: reviews, meta-analyses, systematic evaluations, case studies, conference reports and research protocols.

### 2.3. Data Extraction

Two investigators independently screened and reviewed the articles, with any arising discrepancies resolved by a third investigator.

The following data were extracted from the articles that were finally included in the study:

(1) Basic information: surname of first author, date of publication, type of study, country.
(2) Characteristics of the sample: sample size and age group.
(3) Information on the COVID-19 vaccine: type and dose.
(4) Outcome of morbidity after COVID-19 vaccination: total number of individuals and number of men and women with morbidity after different doses of vaccine. The data we extracted are shown below.

### 2.4. Risk of Bias Assessment

Two investigators independently assessed the quality of included studies using the Newcastle Ottawa Scale (NOS) [19], as used for cohort studies, consulting a third author in the absence of consensus. We categorized studies as low (7–9 stars), moderate (5–6 stars), and high risk of bias (0–4 stars), and included studies with scores ≥7 for meta-analysis. Our assessment of the quality and risk of the literature is shown below.

### 2.5. Data Synthesis and Analysis

The incidence of confirmed myocarditis or pericarditis was extracted from each report, as well as the difference between the first and second dose, and the difference between males and females. Statistical heterogeneity between studies was assessed using I-squared. Because significant heterogeneity was detected, a random-effects model was applied and a meta-analysis was performed to calculate the combined RR (risk ratio) and 95% confidence interval. Stata (version 15.1) was used to analyze the data.

## 3. Results

### 3.1. Characteristics of Included Studies

We identified 152 potentially eligible articles in the PubMed, Web of Science and Embase databases. After deleting duplicate records using Endnote X8, 130 records remained. The majority of articles (*n* = 97) did not meet the inclusion criteria and were excluded after reviewing their titles, abstracts and full texts. A total of 33 articles were screened for eligibility and reviewed for full text, and finally, 7 articles were included in the meta-analysis, including 6 retrospective cohort studies and 1 prospective cohort study.

Of the studies analyzing prevalence, two studies included children and adolescents from China (578,979), two studies from Israel (848,385), one study from Denmark (261,334) and one study from South Korea (444,313). Of the studies analyzing the prevalence in men and women, two studies were from Israel (356,268 male subjects and 369,016 female subjects).

Participants received the BNT162b2 mRNA COVID-19 vaccine in four studies (two studies from Israel, one study from China and one study from Korea); the Pfizer Biologics mRNA COVID vaccine in one study from Denmark; the Comirnaty vaccine in one study in a Chinese population; and one vaccine from a Chinese study is unknown. The risk of bias was low in all studies, indicating high quality.

### 3.2. Incidence of Myocarditis or Pericarditis after the First/Second Dose of the Vaccine

Figures S2 and S3 summarize the incidence of myocarditis or pericarditis after the first and second doses of the vaccine. For the first dose of a COVID-19 vaccine, the combined effectiveness of infection was 0.0022% (95% CI: 0.0010–0.0034%), *p* = 0.000, I-squared = 81.2%, with significant heterogeneity. For the second dose of a COVID-19 vaccine, the combined effectiveness was 0.0107% (95% CI: 0.0059–0.0155%), *p* =0.000, I-squared = 91.1% with significant heterogeneity.

### 3.3. Incidence Risk Relative to the Second and First Doses

Figure S4 summarizes the relative risk of incidence of myocarditis or pericarditis after the first and second doses. The relative risk for the first dose relative to the second dose corresponds to an RR = 5.53 (95% CI: 3.01–10.16) *p* = 0. 114, I-squared = 46.3% with confidence in the data.

### 3.4. Male and Female Relative Risk Incidence of Myocarditis or Pericarditis

Figure S5 summarizes the rate for males relative to females after the second dose of vaccination as RR = 13.91 (95% CI: 4.30–44.95), *p* = 0.851, I-squared = 0.00% and there is confidence in the data.

## 4. Discussion

To our knowledge, this is the first meta-analysis to assess the occurrence of myocarditis or pericarditis following COVID-19 vaccination in adolescents. After screening, seven studies in adolescents, aged 12–19 years, six retrospective cohort studies and one prospective cohort study, were included in this paper. In terms of post-vaccination myocarditis or pericarditis, our study showed that the incidence rates in the adolescent cohort after the first and second doses of the vaccine were 0.0022% and 0.0107%, the ratio of incidence rates for the second dose relative to the first dose was RR = 5.53, and the ratio of incidence rates for males relative to females after the second dose of the vaccine was RR = 13.91. In the pre-COVID-19 era, vaccine-associated pericarditis accounted for 0.1% of the 620,195 reports submitted to the Vaccine Adverse Event Reporting System (VAERS) between 1990 and 2018 [20]. Case reports of myocarditis associated with COVID-19 vaccine were first published in March 2021, and since then, understanding this new adverse event of the vaccine has been a challenge due to a lack of comprehensive data [21]. This meta-analysis summarizes seven retrospective cohort studies that systematically analyzed the incidence and relative risk of myocarditis/pericarditis associated with the first versus second dose of the vaccine in adolescent populations, as well as the relative risk of incidence in males versus females.

The incidence of myocarditis and pericarditis after the first and second doses of the vaccine in the adolescent population is also of great concern affecting adolescent vaccination [22,23,24,25]. We included seven cohort studies for incidence after the first dose [26,27,28,29,30,31,32], which was 0.0022% (95% CI 0.000995–0.003394). We included five cohort studies [27,28,30,31,32] for incidence after the second dose, which was 0.0107% (95% CI: 0.0059–0.0155%). There was a large heterogeneity in the results. Meta-regression analysis excluded the following sources of heterogeneity: number of cases, total number included, country, age and diagnostic criteria. The overall average incidence rate was 1.69 cases per 100,000 person-years. We also looked for epidemiological studies of pre-pandemic myocarditis/pericarditis to compare with the incidence rates in our study. In a Korean study, the incidence of myocarditis/pericarditis in adolescents aged 12–17 years was 1.22/1000 hospitalizations from 2010 to 2019 [33]. In a national study in Finland, the overall incidence of myocarditis in children aged ≤15 years was 1.95/100,000 person-years from 2004 to 2014 [34]. The incidence of pericarditis/myocarditis was not significantly higher after the first or second dose of the vaccine relative to the incidence of pericarditis/myocarditis before vaccination. Although the mechanism is uncertain, this conclusion has been reached in several other studies [35,36,37,38]. ACIP conducted an evaluation using a method previously used to assess the benefit–risk balance of mRNA vaccines in adolescents, comparing the benefits of vaccination in adolescents (prevention of COVID-19 infection and other diseases) with the risks (total number of people with myocarditis after vaccination) [39]. The overall result was that the benefits outweighed the risks [39]. Overall, the risk of harm from myocarditis to adolescents with myocarditis or pericarditis is small, and active vaccination is still needed in the context of an epidemic.

Regarding the difference in morbidity after the first and second doses of the vaccine, five studies were included for relative risk analysis [27,28,30,31,32] and the morbidity ratio for the second dose versus the first dose was RR = 5.53 (95% CI: 3.01–10.16). This was also supported by other studies that showed that a second dose of a COVID-19 vaccine significantly increased the risk of myocarditis/pericarditis relative to the first dose. A case–control study from Hong Kong found that the risk of myocarditis/pericarditis was mainly seen after the second dose of a BNT162b2 vaccine rather than after the first dose [40]. A study from the USA found that the number of illnesses was higher after the second vaccination [39,41]. An observational study based on VigiBase, the World Health Organization’s global database of individual case safety reports, found that the risk of disease increased significantly after the second dose. However, the disease was mild and would heal well with timely treatment [36]. The above indicates the need to monitor health status after a second vaccine dose in order to facilitate timely diagnosis and treatment. Some regions have suggested that the second dose policy could be delayed if the local epidemic is stable, in order to mitigate the high incidence of disease resulting from the second dose (insert), but this suggestion warrants further validation.

In addition to this, several studies have reported different risks of myocarditis and pericarditis in adolescent males and females following COVID-19 vaccination. After screening, two studies in children and adolescents aged 12–19 years were included in this part of our report. Two retrospective cohort studies were conducted by DrorMevorach et al. in 2021 and 2022, respectively [31,32]. After analysis, our study showed that the overall incidence of acute myocarditis and pericarditis after the second dose of the vaccination was 41/356309 in adolescent males and 3/369019 in adolescent females with an RR = 13.91 (95% CI, 4.30–44.95) for males relative to females. This means that this disease process is different in terms of sex, with males being more likely to develop myocarditis and pericarditis after a second dose of a COVID-19 vaccine. Similarly, many reviews have spoken of boys being at greater risk of developing myocarditis/pericarditis after vaccination. Supriya Jain conducted a retrospective multicenter study in 16 hospitals in the USA, where the mean age of the 63 patients diagnosed with C-VAM was 15.6 ± 1.8 years and 58 patients (92%) were male [42]. Jonathan Yap et al. conducted a study of a review of the HSA vaccine adverse event reporting system up to 25 July 2021 and showed that the highest incidence of pericarditis was in males aged 12–19 years, with an incidence rate of 1.11 cases per 100,000 doses [43]. In conclusion, many studies have shown that the incidence of myocarditis and pericarditis after a second dose of the vaccine is higher in males than in females among adolescents, further supporting the results of our meta-analysis. There is no clear pathophysiological mechanism for the explanation of this phenomenon. This may be related to hormone-mediated signaling. Testosterone has the ability to reduce anti-inflammatory immune cells while enhancing the immune response of T helper 1 cells. Estrogen, in contrast, reduces cell-mediated immune responses by suppressing pro-inflammatory T cells [44]. We recommend that male adolescents pay more attention to their adverse reactions after vaccination and that they be treated promptly.

However, our study also had some limitations. Firstly, the limited number of subjects used in our study, the different diagnostic criteria for myocarditis/pericarditis and the fact that the study subjects had different study objectives and study methods all contributed to some bias in the results. When assessing prevalence in men and women, we found too few relevant studies worldwide, making the results potentially biased. Our meta-analysis provides a great tool for enhancing global adolescent vaccination, as our study shows a lower risk of myocarditis/pericarditis prevalence after vaccination. It also provides better post-vaccination recommendations, such as prompt observation of one’s adverse symptoms after a second vaccination dose or after male adolescents are vaccinated, so that a timely diagnosis and treatment can lead to better healing. It also provides clinical direction for doctors to consider the diagnosis of myocarditis/pericarditis in patients who have received a COVID-19 vaccine, especially in men after a second dose of a vaccine, as well as providing ideas for vaccine developers to produce a vaccine that will reduce the risk of myocarditis/pericarditis after a second dose or in men.

## 5. Conclusions

For the current study, there was no significant increase in the incidence of myocarditis/pericarditis after COVID-19 vaccination in adolescents and symptoms were mild. However, after the second vaccine dose in male adolescents, the risk of morbidity was significantly increased relative to the first dose and in female adolescents. We recommend that male adolescents and those who have received a second vaccine dose should be promptly monitored for adverse reactions and treated promptly for early recovery as the symptoms are mild. Studies on myocarditis/pericarditis associated with COVID-19 vaccines in adolescents remain scarce and useful data are limited. Further clinical trials and basic research are needed to explore this.

## Data Availability

Data can be obtained by contacting the corresponding author.

## Supplementary Materials

The following supporting information can be downloaded at: https://www.mdpi.com/article/10.3390/vaccines10081316/s1. Table S1: Search strategy, Table S2: Inclusion of research articles, Table S3: Risk of Bias Assessment, Figure S1: Flow chart of article screening, Figure S2: Incidence of myocarditis or pericarditis after the first dose of a vaccine, Figure S3: Incidence of myocarditis or pericarditis after the second dose of a vaccine, Figure S4: Incidence risk relative to the second and first doses, Figure S5: Male and female relative risk incidence of myocarditis or pericarditis.
