# Supplementary material for: Myocarditis or Pericarditis Following the COVID-19 Vaccination in Adolescents: A Systematic Review
Source: Vaccines (Basel). 2022 Aug 15;10(8):1316. doi: 10.3390/vaccines10081316 (PMC9412657; doi:10.3390/vaccines10081316)
Supplement: Supplementary file 1 [file vaccines-10-01316-s001.zip › vaccines-1812262-supplementary.pdf]

Table S1. Search strategy.

Databases	Detailed Search Strategy
Pubmed	("vaccines" [MeSH Terms] OR "Vaccination" [MeSH Terms] OR "Vaccine" [Title/Abstract] OR "Vaccination" [Title/Abstract] OR "active immunization" [Title/Abstract]) AND ("COVID-19" [MeSH Terms] OR "SARS-CoV-2" [MeSH Terms] OR "COVID-19" [Title/Abstract] OR "SARS-CoV-2" [Title/Abstract] OR "2019 novel coronavirus" [Title/Abstract] OR "SARS-CoV-2" [MeSH Terms] OR "COVID19" [Title/Abstract] OR "2019-nCoV" [Title/Abstract] OR "coronavirus disease 2019" [Title/Abstract] OR "coronavirus disease 2019 virus" [Title/Abstract] OR "sars coronavirus 2" [Title/Abstract] OR "coronavirus disease 19" [Title/Abstract] OR "sars cov 2 virus" [Title/Abstract]) AND ("Child" [MeSH Terms] OR "Infant" [MeSH Terms] OR "Adolescent" [MeSH Terms] OR "Child" [Title/Abstract] OR "Children" [Title/Abstract] OR "Childhood" [Title/Abstract] OR "Infant" [Title/Abstract] OR "Adolescent" [Title/Abstract] OR "Adolescence" [Title/Abstract] OR "Teenager" [Title/Abstract] OR "Youth" [Title/Abstract]) AND ("Myocarditis" [MeSH Terms] OR "cardiomyopathies" [MeSH Terms] OR "Cardiomyopathy" [Title/Abstract] OR "Myocarditis" [Title/Abstract] OR "Pericarditis" [MeSH Terms] OR "Pericarditis" [Title/Abstract])
Wed of science	TS = ((vaccine OR vaccination OR "active immunization" OR "active immunizations") AND ("COVID-19" OR "SARS-CoV-2" OR "2019 novel coronavirus" OR "2019 novel coronavirus" OR COVID19 OR "2019-nCoV" OR "coronavirus disease 2019" OR"SARS Coronavirus 2"OR "Coronavirus Disease-19" OR "SARS CoV 2 Virus" OR "Severe acute respiratory syndrome coronavirus 2") AND (child OR infant OR juvenile OR children OR childhood OR adolescence OR adolescent OR teenager OR youth) AND (myocarditis OR cardiomyopathy OR pericarditis))
Embase	('vaccine'/exp OR 'vaccination'/exp OR 'active immunization'/exp OR 'vaccine' OR 'vaccination' OR 'active immunization':ab,ti) AND ('coronavirus disease 2019'/exp OR 'severe acute respiratory syndrome coronavirus 2'/exp OR 'covid-19'/exp OR 'sars-cov-2'/exp OR '2019 novel coronavirus'/exp OR 'covid19'/exp OR '2019-ncov'/exp OR 'sars coronavirus 2'/exp OR 'coronavirus disease-19'/exp OR 'covid-19' OR 'sars-cov-2' OR '2019 novel coronavirus' OR '2019 novelcoronaviruses' OR 'covid19' OR '2019-ncov' OR 'coronavirus disease 2019' OR 'sars coronavirus 2' OR 'coronavirus disease-19' OR 'sars cov 2 virus':ab,ti) AND ('adolescent'/exp OR 'child'/exp OR 'juvenile'/exp OR 'child':ab,ti OR 'infant':ab,ti OR 'juvenile':ab,ti OR 'children':ab,ti OR 'childhood':ab,ti OR 'adolescence':ab,ti OR 'adolescent':ab,ti OR 'teenager':ab,ti OR 'youth':ab,ti) AND ('myocarditis'/exp OR 'cardiomyopathies'/exp OR 'pericarditis'/exp OR 'myocarditis':ab,ti OR 'cardiomyopathies':ab,ti OR 'pericarditis':ab,ti)

Table S2. Included study articles.

**First author**	**Publication time**	**Study design**	**Country of population**	**Type of COVID-19 vaccine**	**Age of population (years old)**	**Number of people vaccinated with 1st Dose COVID-19**	**Number of people with myocarditis/pericarditis after 1st Dose**	**Number of people vaccinated with 2nd Dose COVID-19**	**Number of people with myocarditis/pericarditis after 2nd Dose**	**male Number of people vaccinated with 2nd Dose COVID-19**	**male Number of people with myocarditis/pericarditis after 2nd Dose**	**female Number of people vaccinated with 2nd Dose COVID-19**	**female Number of people with myocarditis/peicarditis after 2nd Dose**	**Diagnostic criteria**
Dror Mevorach	2022	retrospective cohort study	Israeli	BNT162b2	12-15	404,407	1	326,463	13	157,153	11	169,310	1	*International Classification of*

														Diseases, 10th Revision(ICD-10) and Brighton Collaboration
Dror Mevorach	2021	retrospective cohort study	Israeli	BNT162b2	16-19	443,978	3	398,821	32	199,115	30	199,706	2	*International Classification of Diseases, 9th Revision*(ICD-9) and Brighton Collaboration
Francisco Tsz Tsun Lai	2022	population-based retrospective cohort study	China	BNT162b2	12-18	139,482	8	120,830	30	/	/	/	/	*International Classification of Diseases, Ninth Revision* (ICD-9) and International Classification of Primary Care (ICPC)
Gilbert T Chua	2021	population cohort study	China	Comirnaty vaccine	12-17	178,163	6	/	27	/	/	/	/	Cardiovascular Injury-Coalition for Epidemic Preparedness Innovations (CEPI) and the Brighton Working Group
Ulrikka Nygaard	2021	prospective population-based cohort study	Danish	Pfizer-Biotech mRNA COVID vaccine	12-17	261,334	15	/	/	/	/	/	/	Hospital diagnosis
Xue Li	2022	cohort study	China	/	12-17	224,358	7	162,528	36	/	/	/	/	*International Classification of Diseases, Ninth Revision*(ICD-9), Clinical Modification (422.x and 429.0)

Young June Choe	2022	retrospective cohort study	South Korea	BNT162b2	12-18	444,313	8	442,025	19	/	/	/	/	/

Table S3. Risk of bias assessment.

Quality assessment of cross-sectional study by AHRQ												
**First author and Publication time**	**Define the source of information (survey, record review)**	**Whether the representativeness of the exposure cohort is truly or somewhat representative of the average incidence of post-vaccination myocarditis or pericarditis in the community**	**Whether the non-exposed group is from the same community as the exposed group**	**Exposure is determined through - safety records (e.g. surgical records) or structured interviews**	**Prove that the result of interest did not exist at the beginning of the study**	**Comparability of cohorts based on design or analysis was controlled for studies of the incidence of post-vaccine myocarditis or pericarditis, or for any other factor.**	**The assessment of outcomes is an independent blind assessment or recorded contact**	**Is the follow-up period long enough for the outcome to occur**	**If applicable, explain how missing data were handled in the analysis**	**Adequacy of cohort follow-up, e.g. complete follow-up (all subjects included) or subjects lost to follow-up are unlikely to introduce bias, e.g. the number lost is small (≥60% follow-up)**	**Total score**	**Risk of bias**
Dror Mevorach 2022	0	1	0	1	1	1	1	0	1	1	7	Low risk of bias
Dror Mevorach 2021	1	1	0	1	1	1	1	1	1	1	9	Low risk of bias
Francisco Tsz Tsun Lai 2022	1	1	1	1	1	1	1	1	0	1	9	Low risk of bias
Gilbert T Chua 2021	1	1	0	0	1	1	1	1	1	1	8	Low risk of bias
Ulrikka Nygaard 2021	1	1	0	1	1	1	1	0	1	0	7	Low risk of bias
Xue Li 2022	1	1	1	0	1	1	1	1	1	0	8	Low risk of bias
YoungJune Choe 2022	1	1	0	0	1	1	1	1	0	1	7	Low risk of bias

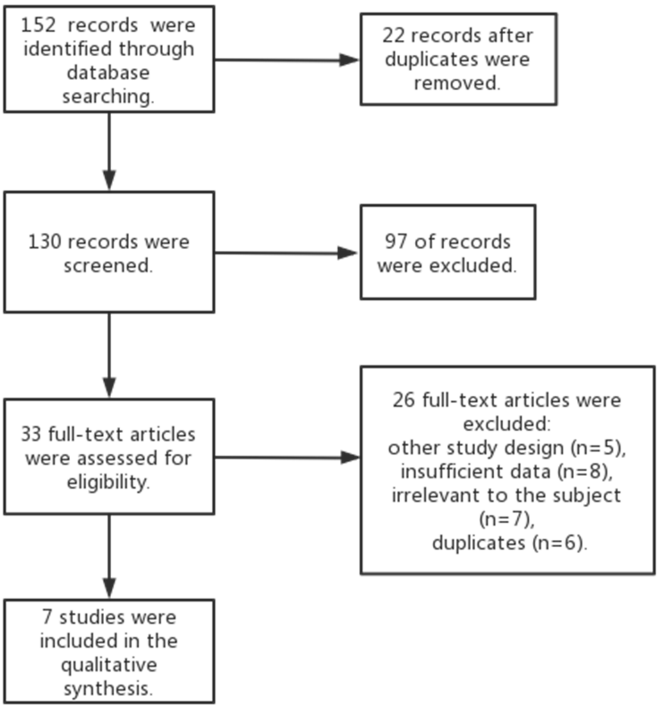

Figure S1. Flow chart of article screening.

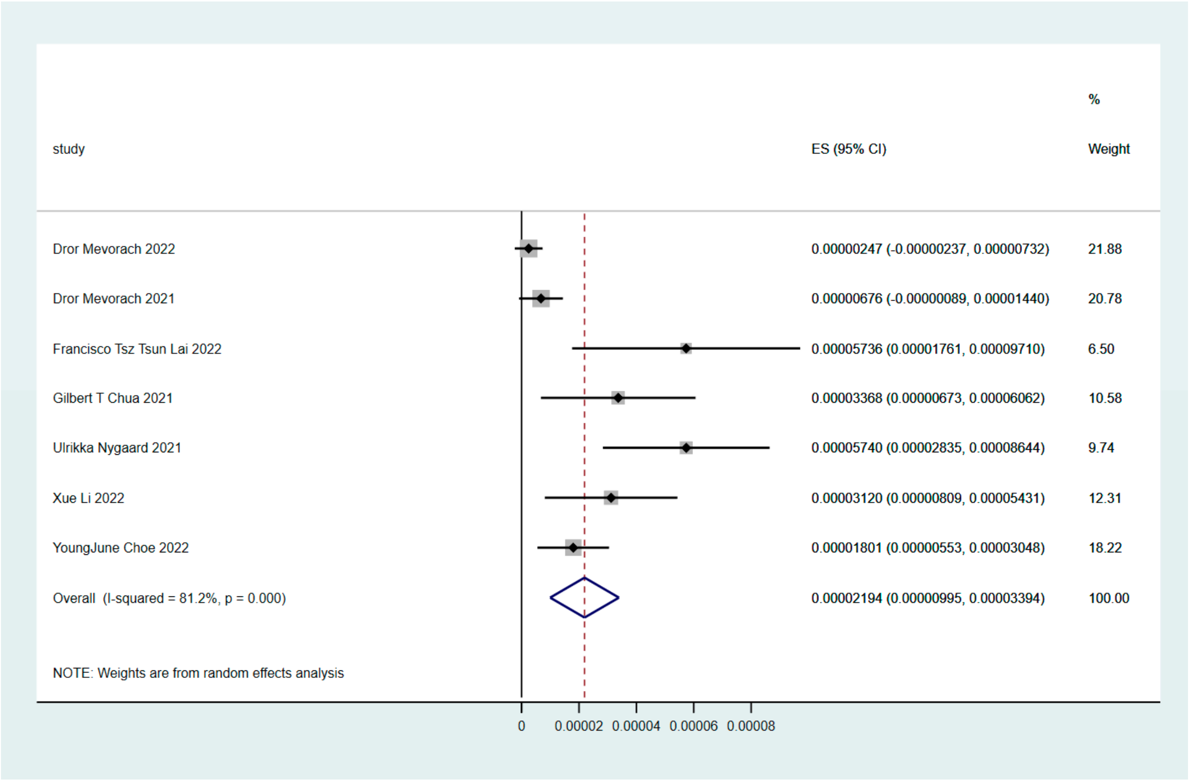

Figure S2. Incidence of myocarditis or pericarditis after the first dose of vaccine.

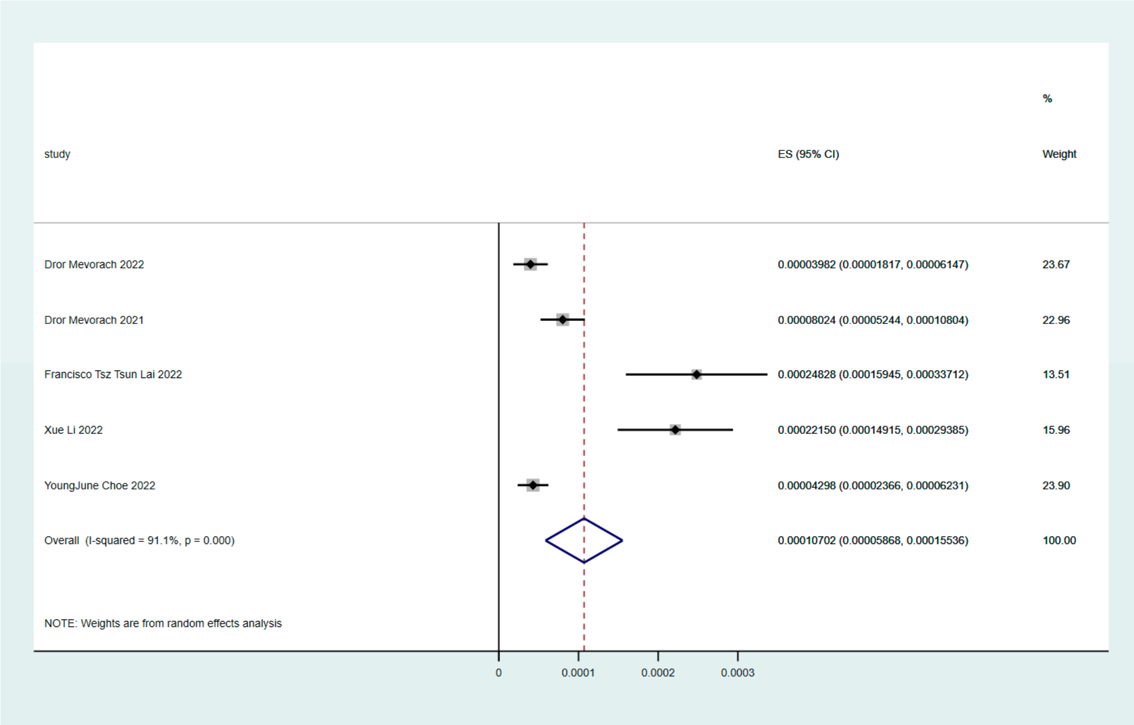

Figure S3. Incidence of myocarditis or pericarditis after the second dose of vaccine.

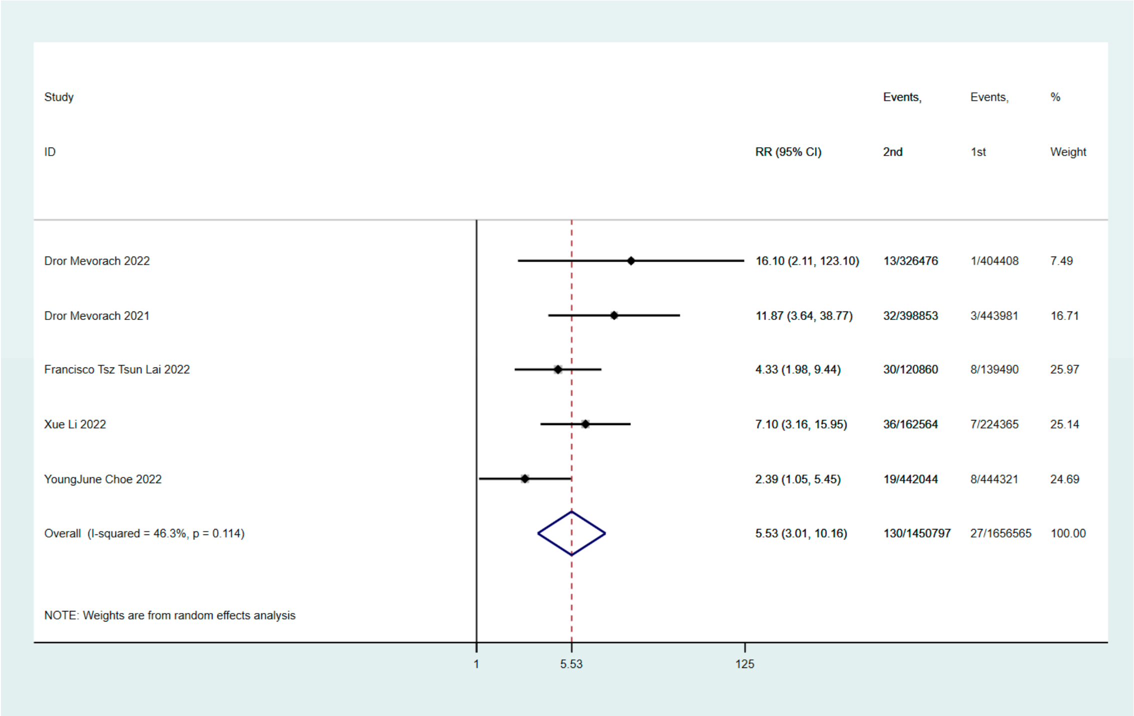

Figure S4. Incidence risk relative to the second and first doses.

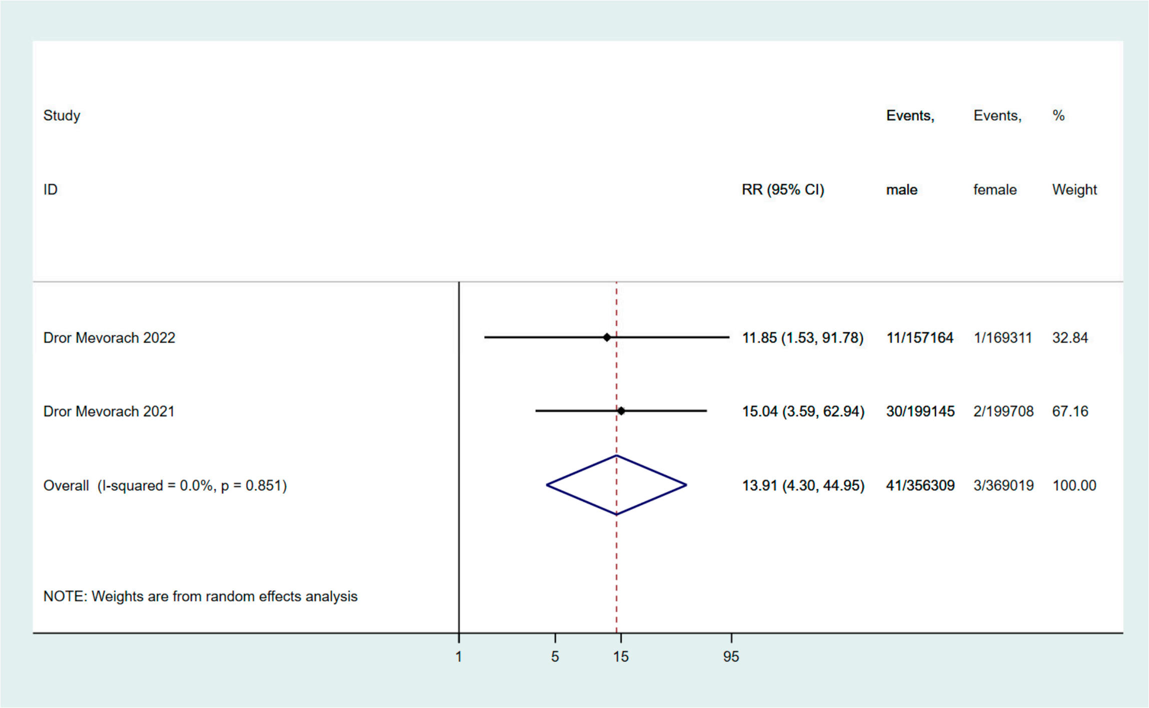

Figure S5. Male and female relative risk incidence of myocarditis or pericarditis.
